# Assessment of Immune Response Following Dendritic Cell-Based Immunotherapy in Pediatric Patients With Relapsing Sarcoma

**DOI:** 10.3389/fonc.2019.01169

**Published:** 2019-11-14

**Authors:** Lenka Fedorova, Peter Mudry, Katerina Pilatova, Iveta Selingerova, Jana Merhautova, Zdenek Rehak, Dalibor Valik, Eva Hlavackova, Dasa Cerna, Lucie Faberova, Pavel Mazanek, Zdenek Pavelka, Regina Demlova, Jaroslav Sterba, Lenka Zdrazilova-Dubska

**Affiliations:** ^1^Department of Pharmacology, Faculty of Medicine, Masaryk University, Brno, Czechia; ^2^Department of Laboratory Medicine, Masaryk Memorial Cancer Institute, Brno, Czechia; ^3^Regional Centre for Applied Molecular Oncology, Masaryk Memorial Cancer Institute, Brno, Czechia; ^4^Department of Pediatric Oncology, University Hospital and Faculty of Medicine, Masaryk University, Brno, Czechia; ^5^Department of Nuclear Medicine, Masaryk Memorial Cancer Institute, Brno, Czechia; ^6^International Clinical Research Center, St. Anne's University Hospital, Brno, Czechia

**Keywords:** dendritic cells, anticancer immunotherapy, dendritic-cell (DC)-based vaccine, pediatric sarcoma, academic clinical trials, immunomonitoring, personalized medicine

## Abstract

Monocyte-derived dendritic cell (DC)-based vaccines loaded with tumor self-antigens represent a novel approach in anticancer therapy. We evaluated DC-based anticancer immunotherapy (ITx) in an academic Phase I/II clinical trial for children, adolescent, and young adults with progressive, recurrent, or primarily metastatic high-risk tumors. The primary endpoint was safety of intradermal administration of manufactured DCs. Here, we focused on relapsing high-risk sarcoma subgroup representing a major diagnosis in DC clinical trial. As a part of peripheral blood immunomonitoring, we evaluated quantitative association between basic cell-based immune parameters. Furthermore, we describe the pattern of these parameters and their time-dependent variations during the DC vaccination in the peripheral blood immunograms. The peripheral blood immunograms revealed distinct patterns in particular patients in the study group. As a functional testing, we evaluated immune response of patient T-cells to the tumor antigens presented by DCs in the autoMLR proliferation assay. This analysis was performed with T-cells obtained prior to DC ITx initiation and with T-cells collected after the fifth dose of DCs, demonstrating that the anticancer DC-based vaccine stimulates a preexisting immune response against self-tumor antigens. Finally, we present clinical and immunological findings in a Ewing's sarcoma patient with an interesting clinical course. Prior to DC therapy, we observed prevailing CD8+ T-cell stimulation and low immunosuppressive monocytic myeloid-derived suppressor cells (M-MDSC) and regulatory T-cells (Tregs). This patient was subsequently treated with 19 doses of DCs and experienced substantial regression of metastatic lesions after second disease relapse and was further rechallenged with DCs. In this patient, functional *ex vivo* testing of autologous T-cell activation by manufactured DC medicinal product during the course of DC ITx revealed that personalized anticancer DC-based vaccine stimulates a preexisting immune response against self-tumor antigens and that the T-cell reactivity persisted for the period without DC treatment and was further boosted by DC rechallenge.

**Trial Registration Number**: EudraCT 2014-003388-39.

## Introduction

Patients with relapsed or refractory Ewing's sarcoma have a very poor prognosis. No substantial improvement has been achieved in the therapy of sarcoma patients in the last two decades despite research, and long-term survival is still <25%. Immunotherapeutic approaches including antigen-presenting cell-based vaccines have been employed as single agent or as part of combination strategies having been substantiated by a report on immunogenicity of Ewing's sarcoma with specific translocation resulting in EWS/FLI1 fusion. Following dendritic cell (DC) vaccine with untreated autologous lymphocytes, 39% of patients had measurable immune response against a neopeptide derived from the fusion gene (1). Promising results were reported after CD25+ regulatory T-cell depletion of an autologous lymphocyte infusion product augmented with interleukin (IL)-7, where immune reconstitution correlated with an improved survival of 63% in Ewing's sarcoma and rhabdomyosarcoma (2). Immunocompetent CD8+ T lymphocytes were observed within the tumor microenvironment of metastases after DC immunotherapy (ITx) but without direct cytotoxic efficacy probably due to expression of PD-1 on lymphocytes and PD-L1 on tumor cells (3). Such immune suppression could be bypassed using recently developed anti-PD-1 and anti-PD-L1 agents, demonstrating improved survival in several malignancies, including anecdotal cases of sarcomas (4, 5).

Proper antigen presentation has a key role in directing the immune system to attack tumor cells by targeting tumor-associated antigens. We manufacture fully personalized monocyte-derived DC-based vaccines that are evaluated in an academic investigator-initiated clinical trial for children, adolescents, and young adults with progressive, recurrent, or primarily metastatic high-risk tumors (EudraCT 2014-003388-39). As a part of clinical and research evaluation of patients, we performed DC characterization, peripheral blood immunomonitoring during DC treatment, and *ex vivo* assessment of T-cell cytotoxic function pre- and post-DC treatment. During peripheral blood immunomonitoring, we quantified circulating immune cells to evaluate both positive and negative players in cancer surveillance and eradication. We focused on absolute lymphocyte count (ALC) and neutrophil-to-lymphocyte ratio (NLR). Both parameters are associated with the number of lymphocytes as key players in the immune response to tumors. Additionally, NLR reflects the number of neutrophils that is a negative prognostic factor often related to paraneoplastic immune response. The peripheral blood lymphocyte compartment contains conventional αβ TCR+ T-cells, B-cells, natural killer (NK) cells, and also minor specific effector and regulatory cell types, including regulatory T-cells (Tregs), CD56+ CD3+ NKT-like cells (6), γδ T-cells (7), and monocytic myeloid-derived suppressor cells (M-MDSCs). These immune cell subsets constitute the actual clinical immunomonitoring, and their characteristics are reviewed in Supplementary Material 1.

This study focuses on high-risk sarcoma patients representing a major diagnosis in this clinical trial. First, we evaluated quantitative association between basic cell-based immune parameters. Next, we described patterns of these parameters and their time changes during the DC vaccination course in the peripheral blood immunograms. As a functional testing, we evaluated immune response of patient T-cells to the tumor antigens presented by DCs in autoMLR proliferation assay. This analysis was performed with T-cells obtained prior to DC ITx initiation and with T-cells collected after administration of the fifth dose of DCs. Finally, we presented clinical and immunological findings from DC-based ITx after relapse in the case of the Ewing's sarcoma patient.

## Methods

### Clinical Trial Design and Methodology

This nonrandomized, open-label, academic, investigator-initiated, phase I/II clinical trial (EudraCT No. 2014-003388-39) was performed at a single center in Czechia in accordance with the principles of the Declaration of Helsinki and Good Clinical Practice. The protocol was approved by the local ethics committee at the site and by the designated authority of Czechia (the State Institute for Drug Control).

Patients eligible for the clinical trial were children, adolescents, and young adults (1–25 years old) with histologically confirmed refractory, relapsing, or primarily metastatic high-risk tumors; Karnofsky or Lansky score ≥50; life expectancy longer than 10 weeks; and adequate function of bone marrow, kidney, liver, and heart defined as absolute neutrophil count (ANC) ≥0.75 × 10^3^/μl, thrombocytes ≥75 × 10^3^/μl, hemoglobin 80 g/l, estimated glomerular filtration rate (eGFR) ≥70 ml/min/1.73 m^2^, serum creatinine ≤ 1.5-fold upper limit for the appropriate age, bilirubin ≤ 1.5-fold upper limit for the appropriate age, AST and ALT ≤ 2.5-fold upper limit for the appropriate age, ejection fraction ≥50%, and fractional shortening ≥27% assessed by echocardiography. In the case of bone marrow infiltration, ANC had to be ≥0.5 × 10^3^/μl and thrombocytes ≥40 × 10^3^/μl. In the case of liver metastases, AST and ALT must have been ≤ 5-fold upper limit for the appropriate age. Patients must not have had severe ongoing toxicity resulting from any previous treatment. Radiotherapy (RTx), myelosuppressive, and immunosuppressive treatment must have been withdrawn at least 3 weeks before tumor tissue harvesting; the only exception is corticoid treatment of brain edema that was allowed. Myelopoietic growth factors must have been withdrawn at least 7 days before tumor tissue harvesting. Targeted therapy must have been withdrawn at least 7 days for tyrosine kinase inhibitors (TKI) or at least 3-fold half-life of the drug (upper limit 6 weeks) before tumor tissue harvesting. The time interval between autologous transplantation and tumor tissue harvest must have been ≥12 weeks and in the case of allogeneic transplantation ≥26 weeks. Patients with seropositivity to HIV1, HIV2, *Treponema pallidum*, hepatitis B or C, known hypersensitivity to the study medication, an autoimmune disease that was not adequately treated, uncontrolled psychiatric disease, or uncontrolled hypertension were not eligible. Allowed medication prior to monocyte harvest (leukapheresis) was as follows: metronomic chemotherapy (CTx), immune checkpoint inhibitors, and anti-CD20 antibodies are allowed as concomitant medication for any time before leukapheresis. Monoclonal antibodies (except anti-CD20), high-dose CTx, and high-dose corticoids must have been withdrawn at least 3 weeks prior to leukapheresis with the exception of corticoid treatment of brain edema, which was allowed. Since November 2017, amendment of the procedure for monocyte harvest was made, and TKI must have been withdrawn according to their half-life: drugs with short half-life of 3–14 h at least 2 days before leukapheresis (axitinib, dabrafenib, dasatinib, ibrutinib, idelalisib, nintedanib, ruxolitinib, trametinib), drugs with medium half-life of 15–35 h at least 7 days before leukapheresis [alectinib, bosutinib, lapatinib, lenvatinib, nilotinib, osimertinib, pazopanib, ponatinib, regorafenib, and non-tyrosine kinase inhibitor (non-TKI) everolimus], and drugs with long half-life of 36–60 h at least 12 days before leukapheresis (afatinib, ceritinib, erlotinib, gefitinib, imatinib, cabozantinib, crizotinib, sorafenib, sunitinib, vemurafenib, and non-TKI temsirolimus). Myelopoietic growth factors must have been withdrawn at least 7 days before leukapheresis/monocyte harvest. Patients previously treated with DCs were not allowed to enter the trial.

The primary endpoint of the trial was assessment of safety by analysis of incidence of adverse events of special interest (AESI; i.e., allergic reactions grade ≥3, acute or subacute autoimmune organ toxicity symptoms manifesting up to 30 days after administration of the vaccine, injection site reactions grade ≥4, infectious complications grade ≥3). The secondary safety endpoint was incidence of all adverse events assessed in relation to type, seriousness, and causality. Secondary efficacy endpoints were time to progression, overall survival, objective response to treatment at 12 and 24 months, and clinical benefit rate assessment at 6 and 12 months.

Investigational medicinal product (IMP) was administered as an add-on therapy to standard treatment. The dose of IMP contains 2 × 10^6^ DCs in 100 μl of cryopreservation medium. DC-based IMP was administered intradermally every 3 ± 1 weeks, up to 35 doses, to a predefined site on the left or right arm near the axillary lymph node. The evening before administration and two evenings after application, topical imiquimod, toll-like receptor (TLR)-7 agonist, was applied on the injection site as an adjuvant. On the day of administration, the patient had to have adequate bone marrow function (defined in the same way as in the entry criteria described above) and was not allowed the following therapy: more than a week systemically administered corticosteroids except treatment for cerebral or spinal edema (single administration of corticoids due to premedication, treatment of allergic reaction, and substitution treatment in secondary hypocortisolism are allowed), anticoagulants in therapeutical dose (prophylactic doses of low-molecular-weight heparins were allowed), erythropoietin, pegylated granulocyte-stimulating growth factors or other growth factors except for filgrastim, RTx to sites and regional lymph nodes, except radiation for pain control, the interval between vaccine application, and administration of conventional CTx must have been more than 72 h. Complete blood count, biochemical analysis, and immunomonitoring were performed on every patient visit associated with administration of IMP.

### DC Manufacturing and Quality Control

The DC-based vaccine, called MyDendrix, was manufactured under GMP in Clean rooms of the Department of Pharmacology, Faculty of Medicine, Masaryk University. Briefly, mononuclear cells were collected by leukapheresis, and then monocytes were separated by elutriation or adherence to a plastic surface. Harvested monocytes were cultivated with IL-4 and granulocyte-macrophage colony-stimulating factor (GM-CSF) and differentiated into DC. Immature DCs were subsequently exposed to autologous tumor lysate antigens. The preparation of tumor lysate from the patient's tumor obtained during curative surgery or extended biopsy preceded monocyte harvest. Maturation was induced by lipopolysaccharide and interferon-γ. Manufactured DCs were aliquoted into IMP doses, each containing 2 × 10^6^ DCs based on reports (8, 9), cryopreserved in DMSO-containing medium, and stored at −150°C to −196°C. Quality control (QC) of DC-based IMP included viability, cell phenotype, production of IL-12 and IL-10, and stimulation of allogeneic and autologous T-cells to reflect the level of stimulatory properties of DCs. Details on DC-based IMP manufacturing were described in Supplementary Material 2 (8, 10). DCs were stored frozen until the day of administration when a DC dose was shipped on dry ice for administration to a study patient, shortly thawed, and immediately injected intradermally to the patient.

### *Ex vivo* Assessment of Prevaccination and Postvaccination T-Cells

Stimulatory properties of DCs were examined pre- and post-DC treatment by autologous mixed lymphocyte reaction (MLR). Pre-DC ITx lymphocytes were obtained during the manufacturing of DCs of from the elutriation process or adherence of leukapheresis product obtained for separation of monocytes. The number of T-cells in the lymphocyte-rich fraction was quantified by flow cytometry: approximately 10^5^ PBMCs were mixed with 10 μl of anti-CD45-PC7 (clone J33) and anti-CD3-FITC (clone UCHT1, both from Beckman Coulter), incubated 20 min in the dark, and analyzed on an FC500 flow cytometer (Beckman Coulter). PBMCs were aliquoted, cryopreserved in 1,000 μl of Cryostor CS5 (BioLife Solutions), frozen, stored at −150°C to −196°C, and thawed prior to auto-MLR seeding. For post-DC treatment assay, PBMCs were obtained from peripheral blood collected into K3EDTA tube (7 ml, Sarstedt) after application of at least five doses of DCs. Blood was layered onto Histopaque-1077^®^ (Sigma-Aldrich, density 1,077 g/ml) and centrifuged (450 g, 30 min, 20°C, acceleration 3, brake 3). Fractions of mononuclear cells were collected and washed with Hank's Balanced Salt Solution (HBSS, Lonza). 10^7^ PBMCs were cryopreserved in 1,000 μl Cryostor CS5 (BioLife solutions) and stored at −150°C. For pre- and post-DC treatment autoMLR, 10^7^ target lymphocytes were stained with 250 μl 10 μM carboxyfluorescein succinimidyl ester (CFSE, Sigma-Aldrich) and seeded into sterile 96-well culture plate (Sarstedt, TC Plate 96-well, Suspension, F) at 10^5^ cells/well in X-vivo 10 medium (Lonza) containing 5% inactivated human male AB serum (Sigma-Aldrich) at a 1:10 effector:target ratio (10^4^ DC/well), positive control (PC) with phytohemagglutinin (PHA, Sigma-Aldrich) 1 mg/ml HBSS (final concentration 10 μg/ml in MLR), or negative control (NC) with complete X-vivo medium, final volume 200 μl/well. MLR experiments were seeded in triplicates and cultured for 6 days at 37°C/5% CO_2_. Then 2 × 10^4^ cells from each well were stained with CD3-PC7 (clone UCHT1, 10 μl/test, Beckman Coulter) for flow cytometric detection of CFSE fluorescence dilution on CD3+ T-cells. Discrimination for dividing cells was set up using the NC. T-cell proliferation was calculated as follows: [(average % of dividing T-cells in 10:1 MLR)−(average % of dividing T-cells in NC)] × 100/[(average % of dividing T-cells in PC)−(average % of dividing T-cells in NC)].

The medium from autoMLR was centrifuged, and pooled supernatant from triplicates was stored at −20°C until analysis. The concentration of interferon-gamma (IFN-γ), tumor necrosis factor alpha (TNF-α), and IL-17A was measured using a flow cytometric bead assay (BD Biosciences).

### Peripheral Blood Immunomonitoring

Detailed peripheral blood immunomonitoring was performed at baseline (= before DC therapy initiation) and at each DC dose administration. The samples were collected on the day of vaccination just before the application of the vaccine. Blood was collected in a 7.5-ml S-Monovette^®^ tube with K_3_EDTA anticoagulant. Lymphocytes (ALC) and neutrophils (ANC) were measured using a Sysmex XN hematology analyzer. NLR was calculated as ANC/ALC. Immunophenotype was analyzed by multiparameter multicolor flow cytometer and software (Navios, Beckman Coulter). Diagnostic antibodies were purchased from Beckman Coulter, premixed in equal amounts in five cocktails, and stored in the dark at 2–8°C not longer than 7 days: 1/ CD14-PE (RMO52), CD15-KrO (80H5), CD11b-APC (Bear1), CD33-FITC (D3HL60.251), CD45-PB (J33), HLA-DR-PC5 (Immu357); 2/ CD3-FITC (UCHT1), CD4-PB (13B8.2), CD16-PC7 (3G8), CD56-PE (NKH-1); 3/ CD3-FITC (UCHT1), CD4-PB (13B8.2), CD27-AF750, CD45-KrO (J33), CD45RO-ECD (UCHL1), HLA-DR-PC5 (Immu357); 4/ TCR PAN γ/δ-FITC (IMMU510), TCR Vγ9-PC5 (IMMU360), TCR Vδ2-PB (IMMU 389), CD314-APC (ON72); 5/ CD3-FITC (UCHT1), CD4-PC7 (SFCI12T4D11), CD25-PC5 (B1.49.9), CD127-PE (R34.34). Blood (25 μl) was incubated with 10 μl of premixed antibody cocktail for 15 min in the dark at room temperature, hemolyzed by Versalyse^®^ (Beckman Coulter) for 15 min and measured in five flow cytometric assays to detect: (1) M-MDSCs detected as CD45+ CD14+ CD11b+ CD33+ HLA-DR−, and their absolute count was calculated using the number of white blood cells (WBC) measured by the Sysmex XN hematology analyzer; (2) NK cells detected as CD3− CD56+ CD16+, NKT-like cells detected as CD56+CD3+; (3) circulating effector CD8+ T-cells were defined as CD3+ CD8+ CD27–, activated CD8+ T-cells were defined as CD8+ HLA-DR+; (4) γδ T-cell subsets classified as δ2+γ9−, δ2+γ9+, δ2−γ 9+, δ2−γ9− and evaluated for CD314; (5) Tregs defined as CD3+ CD4+ CD25+ CD127−/low+.

### ^18^F-FDG PET/CT Scan

^18^F-FDG PET/CT examination was performed using the hybrid scanner Biograph 64 HR+ (Siemens Erlangen, Germany). CT scan was provided in low-dose CT (25 mAs eff/120 kV). The patient had standard preparation prior to examination, including restriction of physical activity for 12 h, fasting for at least 6 h, capillary glycemia lower than 10 mmol/l (180 mg/dl) prior to ^18^F-FDG administration and peroral hydration with 500–1,000 ml of plain water. ^18^F-FDG was administered at a dose of 262 MBq in study 7/2017 and at a dose of 260 MBq in 1/2018. After an *in vivo* accumulation time of 60 min, whole-body scanning from the proximal third of thighs to the vertex of the skull was performed in both studies. All images were iteratively reconstructed and corrected for attenuation. ^18^F-FDG uptake was assessed visually and also semi-quantitatively in the defined region of interest with calculation of target-to-liver ratios. A target-to-liver ratio higher than 1.0 was considered positive in all evaluated regions.

### Statistical Analysis

Spearman correlation coefficient with significance test was used to measure the strength of the relationship between baseline circulating immune parameters. Graphic visualization of immunograms was performed using radar plot. Non-parametric Wilcoxon test for paired samples was used for analysis of pre- and post-treatment T-cell stimulation. *P*-values <0.05 were considered statistically significant. All statistical analyses were performed with R 3.5.3 software (11).

## Results

### Clinical Trial Progress With Focus on Sarcoma Patients

**Figure 1:**
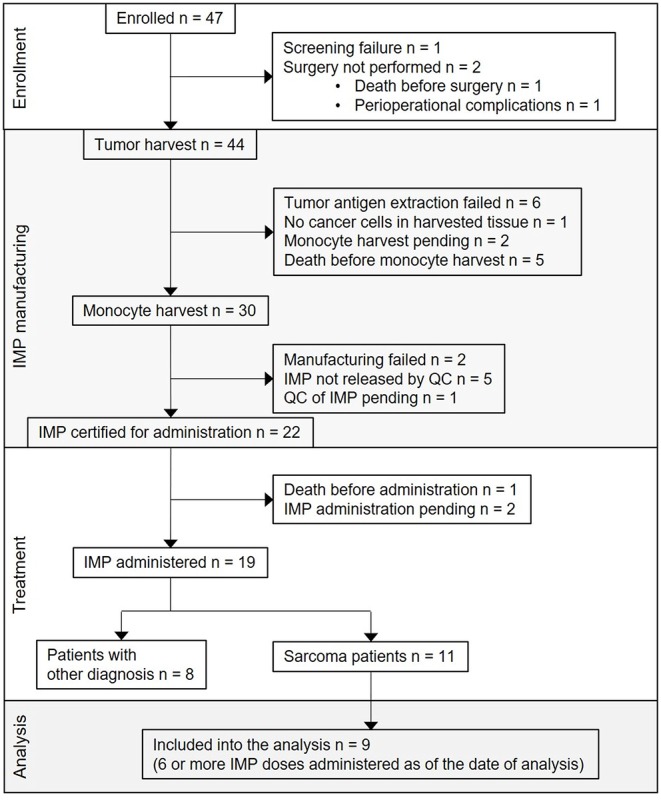
Flow diagram for DC-based immunotherapy trial and study group definition. CONSORT flow diagram showing participant flow through each stage of the trial [enrollment, DC-based investigational medicinal product (IMP) manufacturing, treatment] and the analysis of sarcoma patients study group.

The first subject was enrolled in September 2015. As of May 2019, the clinical trial was still ongoing, but with the accrual suspended. From the overall 47 enrolled patients, 25 (53%) were sarcoma patients. Screening failure occurred in one subject, and tumor harvest was not performed in two subjects. Tumor was harvested in 44 subjects; among them, the harvested tissue contained no cancer cells in one subject, tumor antigen extraction failure presenting as low concentration of protein in tumor lysate in six subjects, participation in the trial ended in five subjects due to disease progression and/or death, monocyte harvest has been pending in two subjects, monocyte harvest and subsequent manufacturing of DC-based IMP was performed in 30 subjects. Of the 30, manufacturing failed in two subjects, IMP did not pass quality control specifications in five subjects (four of them are sarcoma patients) (10), and 22 DC-based IMPs were released for administration to the patients. Of the 22, one subject died before IMP administration, administration has been pending in two sarcoma patients until the completion of high-dose CTx, and DC vaccine was administered to 19 subjects, including 11 sarcoma patients. Of these 11, nine patients received at least six doses of DC-based IMP as of March 2019 and were analyzed in presented immunomonitoring study (Figure 1). The age of sarcoma patients in the study group ranged from 10 to 24 years at the DC ITx initiation (Table 1). Stage of the disease in the study group at the DC ITx initiation was as follows: one (11%) in complete remission, three (33%) subjects in partial remission, one (11%) with stable disease, four (44%) with progressive disease (Table 1). Detail clinical course of disease in nine sarcoma study patients is summarized in Supplementary Material 3.

**Table 1:** Baseline patient characteristics and peripheral blood immune cell levels at dendritic cell (DC) therapy initiation.

**Subject no./**	**Primary diagnosis (primary localization)**	**Stage of the disease and PS at**	**Age at DC ITx init**.	**Baseline cell-based immune parameters at DC ITx initiation**								
**sex**		**DC ITx init**.		
				ALC*	**Eff. CD8+**	**Act. CD8+**	NK*	NKT-like*	GD %*	Tregs*	M-MDSC count*	NLR*
				**10^**6**^/ml**	**%**	**%**	**%**	**%**	**%**	**%**	**10^**6**^/ml**	**Ratio**
KDO-0101/F	Ewing sarcoma (mandible)	2nd CR Karnofsky 100	15 years	**1.9**	73.8	34.8	1.6↓	**4.9**	**2.9**	**5.9**	**0.07**	0.8↓
KDO-0102/F	Osteosarcoma (right distal femur)	PD Lansky 80	10 years	1.3↓	58.9	21.5	**4.6**	**1.9**	**4.6**	2.9↓	**0.07**	**1.2**
KDO-0114/M	Synovial sarcoma (left thigh)	PD Karnofsky 80	15 years	0.2↓	34.3	72.0	0.5↓	**2.9**	1.1↓	**12.0**	0.63↑	19.9↑
KDO-0118/F	Ewing sarcoma (spine C5-Th2)	PR Karnofsky 100	24 years	0.6↓	31.3	11.6	**8.1**	**2.2**	**3.2**	**4.5**	0.25↑	5.2↑
KDO-0119/F	Alveolar rhabdomyo-sarcoma (primum ignotum)	PR Karnofsky 80	13 years	0.6↓	25.1	10.8	**5.8**	**4.6**	**2.6**	**13.4**	**0.24**	**2.7**
KDO-0124/F	Osteosarcoma (right proximal tibia)	2nd mts relapse Karnofsky 100	19 years	0.8↓	73	21.9	**6.4**	**1.5**	**6**	2.9↓	**0.04**	**1.6**
KDO-0131/M	Embryonal rhabdomyosarcoma (pelvis)	PR Karnofsky 70	19 years	0.6↓	94.9	60.5	3.5↓	**14.8**	**3.1**	3.0↓	**0.00**	**1.7**
KDO-0133/M	Osteosarcoma (right proximal femur)	PD Karnofsky 100	24 years	0.9↓	49.1	2.7	**6.5**	**1.5**	**2.8**	3.0↓	0.26↑	**2.9**
KDO-0139/F	Osteosarcoma (left distal femur)	SD Karnofsky 90	22 years	0.5↓	14.73	37.9	4.9↓	**1.2**	0.6↓	**7.9**	0.42↑	10.7↑

Cell-based immune parameters and their age-specific reference range (*if available): ALC, absolute lymphocyte count (reference range^1^ 10 – 16 years 1.4–4.2 × 10^6^/ml, >16 years 1.2–4.1 × 10^6^/ml); NLR, neutrophil-to-lymphocyte ratio (reference range^2^ 1–3); Ef CD8+, circulating effector cytotoxic T-cells (CD27-/CD8+, % of CD8+ T-cells); Act CD8+, activated cytotoxic T-cells (HLA-DR+/CD8+; % of CD8+ T-cells); NK cells, natural killers (reference range^1^ 10–16 years 4–51% of lymphocytes, >16 years 5–49% of lymphocytes); NKT-like, circulating CD3+CD56+ cells (reference range^1^ 10–16 years 0.64–15% of lymphocytes, >16 years 1–18% of lymphocytes); GD-T, gamma-delta T-cells (reference range^1^ 10–16 years 2–17% of lymphocytes, >16 years 0.8–11% of lymphocytes); Treg, regulatory T-cells (reference range^1^ 10–16 years 4–20% of CD4+ T-cells, >16 years 4–17% of CD4+ T-cells); M-MDSC, monocytic myeloid-derived suppressor cells (reference range^3^ 0–0.24 × 10^6^/ml). Numbers in bold refer to the values within the reference range, ↑–above the upper limit of the reference range, ↓–bellow the lower limit of the reference range. ^1^Reference range originated from Schatorje et al. (12). ^2^Estimated from reference ranges for relative differential cell blood count (13). ^3^Own reference value, source group described in Pilatova et al. (13). init., initiation; F, female; M, male.

No immune or infection-related AESIs were reported for all 15 evaluated subjects receiving DC ITx by the date of analysis.

### Peripheral Blood Immunomonitoring of DC-Treated Sarcoma Patients

**Figure 2:**
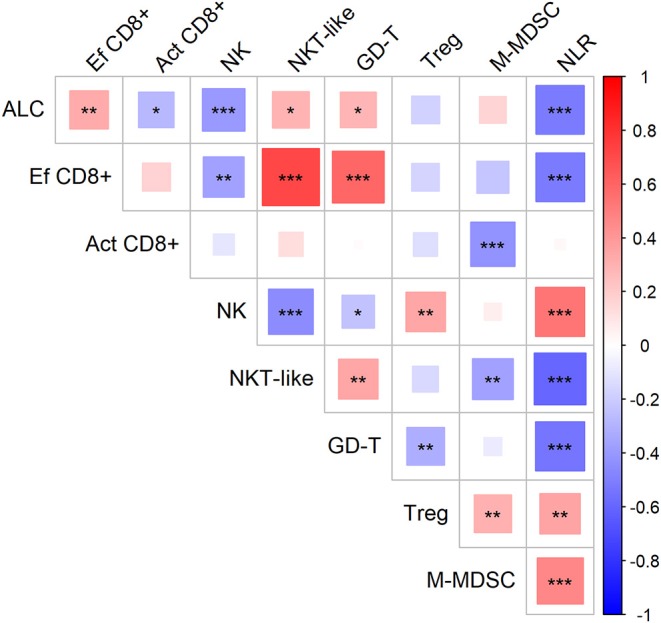
Association of circulating immune markers during the course (from baseline to the sixth dose) of therapy with dendritic cells (DCs) in sarcoma study group. Red—positive correlation, blue—negative correlation; strength of relationship is represented by size of the square and intensity of the color, larger squares with intensified color have stronger relationship; ^*^p < 0.05, ^**^p < 0.01, ^***^p < 0.001; ALC, absolute lymphocyte count (10^6^/ml); NLR, neutrophil-to-lymphocyte ratio; Ef CD8+, circulating effector cytotoxic T-cells (% of CD27− of CD8+ T-cells); Act CD8+, activated cytotoxic T-cells (% of HLA-DR+ of CD8+ T-cells); NK, circulating NK-cells (% of lymphocytes); NKT-like, circulating NKT-like cells (% of lymphocytes); GD-T, γδ T-cells (% of lymphocytes); Treg, regulatory T-cells (% of CD4+ T-cells); M-MDSC, monocytic myeloid-derived supressor cells (10^6^/ml).

First, we evaluated the possible association of cell-based immune parameters in sarcoma patients before DC ITx and during DC treatment, up to six doses of DCs (Figure 2). Based on positive and negative correlations, immune parameters clustered *de facto* into two groups with inverse relation; a group consisting of ALC, proportion of effector cytotoxic T-cells among all T-cells, proportion of CD56+ CD3+ NKT-like cells among lymphocytes, proportion of γδ T-cells among lymphocytes, and an inversely correlated group with neutrophil-to-lymphocyte ratio (NLR), proportion of regulatory T-cells among CD4+ cells, number of M-MDSC, proportion of activated HLA-DR+ CD8+ cells among CD8+ cells, and proportion of CD56+ CD16+ CD3− NK cells among lymphocytes (Figure 2).

**Figure 3:**
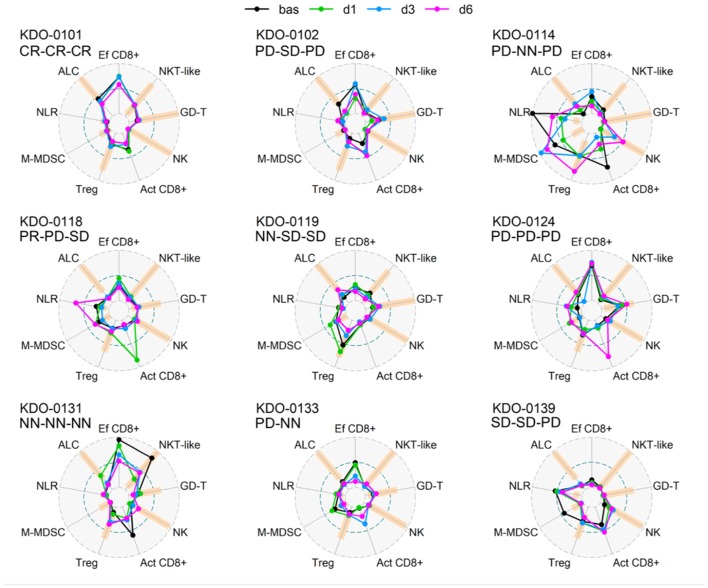
Peripheral blood immunograms of dendritic cell (DC)-treated sarcoma patients. Nine circulating immune parameters are radially arranged with reference ranges shown in orange. Parameters are scaled according to numbers achieved within the entire study group of nine patients. Outer circle (OC, gray dashed) represents the upper limit of the reference range for ALC, NK cells, NKT-like cells, GD T-cells, maximum number reached for the particular marker for Tregs, M-MDSC, and NLR or 100% for Ef CD8+ and Act CD8+; small inner circle (IC, gray dashed) represents zero level; middle circle (MC, pacific blue dashed) represents 50% of OC level. Particular levels are listed for each parameter as follows. ALC, absolute lymphocyte count (reference range^1^ 10–16 years 1.4–4.2 × 10^6^/ml, >16 years 1.2–4.1 × 10^6^/ml; OC: 4.2 10^6^/ml); NLR, neutrophil-to-lymphocyte ratio (reference range^2^ 1–3; OC 19.9); Ef CD8+, circulating effector cytotoxic T-cells (CD27−/CD8+; % of CD8+ T-cells) (OC: 100%); Act CD8+, activated cytotoxic T-cells (HLA-DR+/CD8+; % of CD8+ T-cells) (OC 100%), NK cells (reference range^1^ 10–16 years 4–51% of lymphocytes, >16 years 5–49% of lymphocytes; OC: 51% of lymphocytes); NKT-like, circulating CD3+CD56+ NKT-like cells (reference range^1^ 10–16 years 0.64–15% of lymphocytes, >16 years 1–18% of lymphocytes, OC 18% of lymphocytes); GD-T, γδ T-cells (reference range^1^ 10–16 years 2–17% of lymphocytes, >16 years 0.8–11% of lymphocytes; OC: 17% of lymphocytes); Treg, regulatory T-cells (reference range^1^ 10–16 years 4–20% of CD4+ T-cells, >16 years 4–17% of CD4+ T-cells; OC: 25.3% of CD4+ T-cells); M-MDSC, monocytic myeloid-derived suppressor cells (reference range^3^ 0–0.24 × 10^6^/ml; OC: 0.98 × 10^6^/ml). Baseline levels prior to DC ITx initiation are shown in black and levels at doses d1, d3, d6 are shown in shades of blue. Clinical outcome is shown for each subject at DC ITx initiation, at dose 5, at dose 9. Clinical outcome is abbreviated as follows: CR, complete response; PD, progressive disease; SD, stable disease; NN, non-CR/non-PD; NA, not available. ^1^Reference range originated from Schatorje et al. (12). ^2^Estimated from reference ranges for relative differential cell blood count. ^3^Our user-defined reference value, source group described in Pilatova et al. (13).

Baseline circulating immune parameters in nine sarcoma patients are shown in Table 1. At baseline, eight of nine patients had lymphopenia with mean ALC of 0.81 × 10^6^/ml (Table 1). An exception was patient KDO-0101 (ALC 1.9 × 10^6^/ml) with Ewing's sarcoma whose clinical course and laboratory findings are described later. The proportion of NK cells was low in six of nine patients (median 4.9%, min. 0.5%, max. 8.1%). The proportion of NKT-like cells among lymphocytes was predominantly low (median 2.2%), except for expanded NKT-like cells (14.8% of lymphocytes) in patient KDO-0131. γδ T-cells were low in six of nine patients (median 2.9%, min. 0.6%, max. 6.0%). Based on observed positive and negative association between particular cell-based immune markers, we constructed peripheral blood immunograms with putative anticancer effectors in upper part of an immunogram (namely, total lymphocytes, effector cytotoxic T-cells, CD56+ CD3+ NKT-like cells, γδ T-cells), and on the other hand, cancer-promoting or immunosuppressive actors (namely, NLR, M-MDSC, Tregs) and related factors (activated T-cells and NK cells) in the lower part of an immunogram (Figure 3). In peripheral blood immunograms, we presented baseline values of cell-based immune markers and their level after doses 1, 3, and 6 of ITx with DCs (Figure 3). The peripheral blood immunograms revealed distinct patterns in particular patients in the study group. For instance, we observed “immune-activated” pattern with patient KDO-0101 with Ewing‘s sarcoma who started DC ITx in the second complete remission, ALC was not decreased, effector cytotoxic T-cells represented the majority of circulating T-cells, and NLR and M-MDSC count were low. On the other hand, case KDO-0114 with progressing synovial sarcoma appeared to have an “immune-suppressive pattern” with high NLR, M-MDSC count, Tregs, and low ALC, proportion of effector cytotoxic T-cells, as well as NKT-like and γδ T-cells. Regarding time-dependent variations over the DC vaccination course, we did not observe any consistent trend in the dose-dependent change of levels of evaluated immune system parameters.

### Patient T-Cells *in vitro* Stimulation by DCs Before and After DC Vaccination

**Figure 4:**
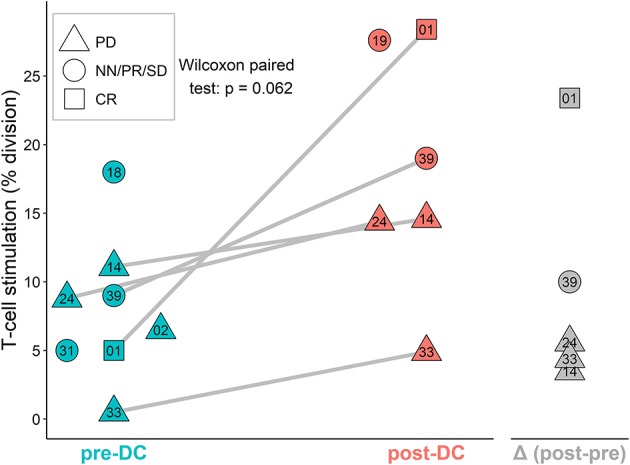
AutoMLR with patients' pre-dendritic cell (DC) and post-DC T-cells stimulated by DC-based investigational medicinal product (IMP). The stimulation is expressed as the percentage of dividing autologous T-cells after incubation with DCs. Pre-DC (blue) refers to the stimulation of patients' T-cells obtained prior to DC-based ITx initiation. Post-DC (red) refers to the stimulation of patients' T-cells obtained after the fifth dose of DC vaccine. The difference (post-DC)−(pre-DC) is shown in gray. The shape of symbols refers to a stage of the disease; PD, progressive disease; CR, complete remission; NN, non-CR/non-PD; PR, partial remission; SD, stable disease. Two-digit numbers refer to the last digits in patients' number (e.g., 01 = KDO-0101, etc.). A pair of pre-DC and post-DC autoMLR in the same patient is linked by a gray line.

The stimulation of sarcoma patient T-cells was examined by MLR proliferation assay with DCs from manufactured IMP and autologous T-cells obtained before DC ITx (pre-DC) and after at least five doses of DCs (post-DC) (Figure 4). The level of auto-MLR ranged from 0.5 to 18% (median 7.7%) with T-cells collected before DC ITx and from 4.9 to 28.4% (median 14.6%) with T-cells obtained after DC vaccination. Paired data with both pre-DC and post-DC were available for five cases, and all exhibited an increase in the T-cell stimulation after DC ITx. We observed the lowest post-DC increase in autologous T-cell stimulation by self-tumor antigens in cases KDO-0114, KDO-0124, and KDO-0133 who started DC treatment in disease progression. On the other hand, the highest increase in the T-cell stimulation with post-DC T-cells was exhibited by patient KDO-0101 who started DC ITx in complete remission of Ewing's sarcoma and remained at least up to ninth dose of DCs in complete remission. This case is described in more detail.

### DC-Based Therapy After Relapse in a Ewing's Sarcoma Patient: Treatment Course and Outcome

**Figure 5:**
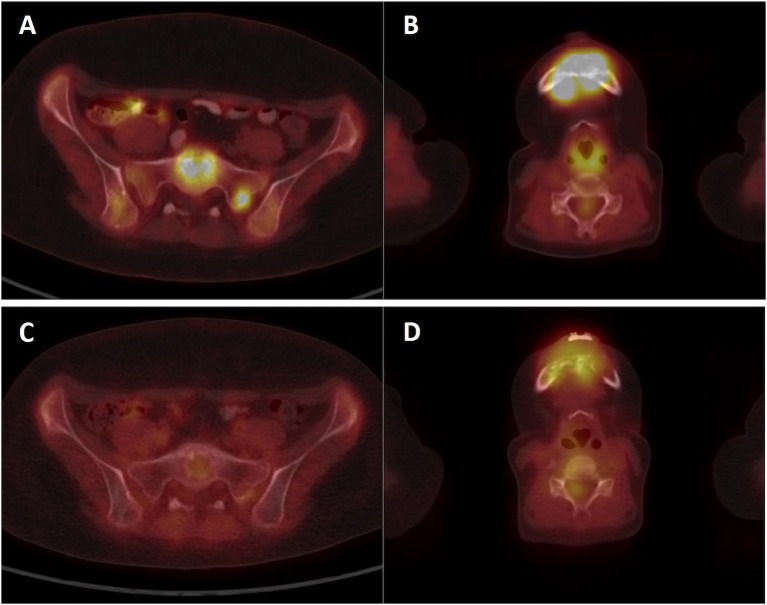
PET/CT imaging of patient KDO-0101. **(A,B)** Examination of patient at second relapse in July 2017 showed ^18^F-FDG-positive osteolytic lesions in the skeleton **(A)** sacrum, sacral base with a target-to-liver ratio of 2.74 and sacral left lateral mass with a target-to-liver ratio of 2.39 **(B)** mandible with a target-to-liver ratio of 4.88. **(C,D)** Control ^18^FDG-PET/CT examination in January 2018 showed a decrease or complete diminishment of ^18^FDG accumulation **(C)** sacrum, sacral base with a target-to-liver ratio of 0.69, and sacral lateral mass with a target-to-liver ratio of 0.66 **(D)** mandible with a target-to-liver ratio of 1.47.

A girl, born 2001, was diagnosed with primary disseminated EWS/FLI-1 positive Ewing sarcoma with a primary tumor in the mandible and skull metastases in December 2011. The patient was treated by protocol EuroEwing 2008, 6x VIDE: vincristine (1.5 mg/m^2^/day; day 1), ifosfamide (3,000 mg/m^2^/day; days 1, 2, 3), doxorubicin (20 mg/m^2^/day; days 1, 2, 3), etoposide (15 mg/m^2^/day; days 1, 2, 3), 1 × VAC: vincristine (1.5 mg/m^2^/day; day 1), actinomycin (0.75 mg/m^2^/day; days 1, 2), cyclophosphamide (1,500 mg/m^2^/day; day 1) from 12/2011 to 10/2012. Surgery was performed in June 2012 with partial resection of primary tumor. Radical resection was not possible due to mutilation. High-dose (HD) CTx treosulphan/melphalan with autologous peripheral blood stem cell transplantation (APBSC) followed in July 2012. Then, the patient underwent RTx of the mandible and parietal bone from September 2012 to November 2012 (34 Gy + 45 Gy), and CTx continued by protocol EuroEwing 2008 with 7× VAC from October 2012 to May 2013. The first complete remission was achieved and lasted until May 2015 when the first relapse occurred in the skull. The patient was enrolled in the DC clinical trial, and the surgically removed tumor from the skull was used as a source of tumor antigens. In the second-line CTx, the patient received vincristine (1.5 mg/m^2^/day; 5 days block), irinotecan (50 mg/m^2^/day; 5 days block), and pazopanib (200 mg/daily). Monocytes were harvested in January 2016, and 35 doses of DC-based medicinal product were manufactured. One week after monocyte separation, palliative RTx on lesions in the skull was started and was performed from January 2016 to February 2016 with a total dose 41 Gy. Subsequently, after recovery from HD CTx and RTx, experimental DC-based ITx (on a biweekly basis) with immunomodulation *via* low-dose cyclophosphamide (26 mg/m^2^/day) started in August 2016. The patient received 19 doses of DCs until the second relapse in 7/2017 with multiple metastases in the skull, pelvis (Figures 5A,B), and lesions in liver. FDG PET positivity without CT scan correlates was noted in the spinal column. Third-line CTx with topotecan (0.75 mg/m^2^; 5 days block), cyclophosphamide (250 mg/m^2^; 5 days block), and zoledronate (4 mg/4 weeks) with concomitant RTx was initiated. Evaluation of response showed stable disease. After three cycles, CTx was stopped due to hematological toxicity. Surprisingly, during the subsequent 4 months without treatment, substantial regression of metastases was noted both on PET/CT scan in 1/2018 (Figures 5C,D) and upon clinical examination of palpable metastases. Fourth-line maintenance metronomic CTx with low-dose vinblastine (3 mg/m^2^/day) and continuing zoledronate (4 mg/dose/4 weeks) was started with rechallenge with DC-based vaccines from the original manufacturing from March 2018 to August 2018. Unfortunately, the partial regression was temporary, and slow continuing progressive disease led to the death of the patient in November 2018.

### DC-Based Therapy After Relapse in a Ewing's Sarcoma Patient: *Ex vivo* Prevaccination and Postvaccination T-Cell Response and Peripheral Blood Immunomonitoring

**Figure 6:**
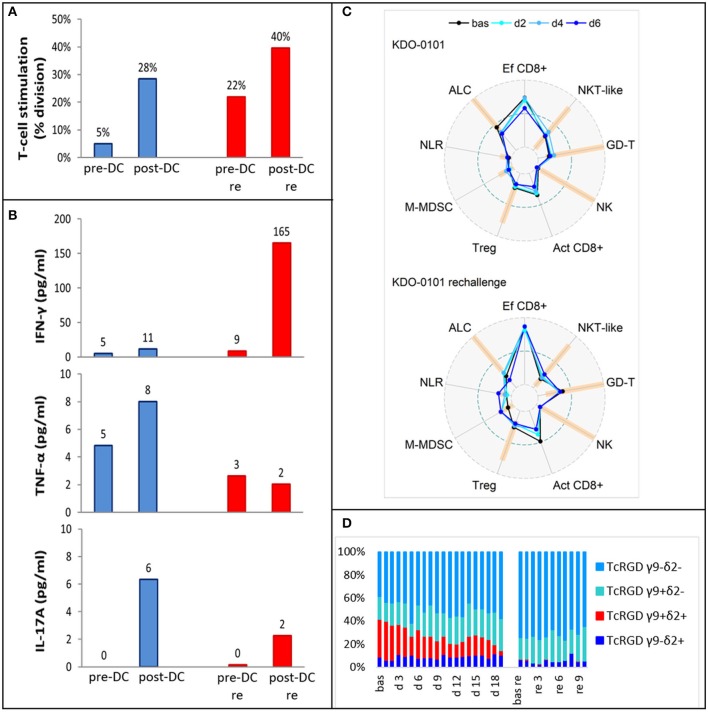
*Ex vivo* functional and peripheral blood immunomonitoring of subject KDO-0101 during first dendritic cell (DC) immunotherapy and its rechallenge. **(A)** Stimulation of T-cells by DCs, reflected by the percentage of division T-cells. **(B)** Production of interferon-γ, tumor necrosis factor (TNF)-α, and interleukin (IL)-17A. **(A,B)** Pre- and post-DC treatment T-cell response was measured i/ (blue) before start of DC administration (pre-DC) and after the fifth dose (post-DC) ii/ (red) after 4 months with no antitumor treatment, before start of DC rechallenge (pre-DC re) and after the fifth rechallenege dose (post-DC re). **(C)** Peripheral blood immunogram from baseline (bas) through doses 2, 4, and 6 in the course of both DC treatment (upper) and DC rechallenge (lower). The layout of immunograms is described in Figure 3. **(D)** Four subtypes of gamma-delta TCR (Vγ9−Vδ2−, Vγ9+Vδ2−, Vγ9+Vδ2+, Vγ9−Vδ2+) in the course of both DC treatment from baseline to dose 19 and DC rechallenge from baseline to dose 10.

Pre-DC treatment T-cell response evaluated by autoMLR as a part of DC quality control resulted in a mean of 5% T-cell division. Post-DC (after the fifth dose) autoMLR exhibited 28% T-cell division (Figure 6A blue). Production of cytokines (IFN-γ, TNF-α, IL-17A) during auto-MLR mildly increased in post-DC compared to pre-DC evaluation (Figure 6B blue). AutoMLR with T-cells collected before restart of DC treatment in February 2018 (after the third-line Ctx with topotecan, cyclophosphamide, and zoledronate with RT and an additional 4 months with no antitumor treatment) exhibited 22% T-cell division and, upon the fifth “rechallenge” dose, 40% T-cell division was observed (Figure 6A red). IFNγ production during autoMLR substantially increased after the fifth dose of DC rechallenge (Figure 6B red). The variations of circulating immune markers exhibited only minor changes at the beginning of both lines of therapy with DCs (Figure 6C). Levels of circulating immune markers at each dose of both lines of DC-based therapy are shown in Supplementary Material 4. At DC rechallenge, an increase in the proportion of circulating effector CD8+ cells and an increase in the proportion of γδ T-cells compared to the initiation of first-line DCs was observed (Figure 6C). In this patient, γδ T-cells were predominantly Vγ9-Vδ2- prior to DC ITx initiation (baseline 39%). Vγ9+Vδ2+ T-cells represented 33% of γδ T-cells, and their proportion decreased during DC Itx, and this γδ subset was almost depleted from circulation after third-line CTx (Figure 6D). In contrast to the Vγ9+Vδ2+ subset, Vγ9-Vδ2- T-cells were predominantly CD314(NKG2D)+ (Supplementary Material 4).

## Discussion

The primary endpoint of the clinical trial investigating anticancer therapy with DCs was the evaluation of treatment safety with interim result from 15 patients of no immune- or infection-related adverse events. Moreover, to gain more information from DC-treated patients, we performed immunomonitoring at baseline and at each DC dose. Collected data will be evaluated in the context of clinical outcomes after completion of the trial.

Here we show that an ALC was positively associated with the proportion of effector CD8+ cytotoxic T-cells out of total T-cells that is reflected by an inversion of the CD4:CD8 ratio and proportion of effector cells CD8+ among total CD8+ cytotoxic T-cells. The proportion of effector CD8+ cytotoxic T-cells among total T-cells was further correlated with the proportion of NKT-like cells and γδ T-cells. Both of these non-classical lymphocyte subsets have been studied and described for their role in cancer surveillance (6, 14, 15). On the other hand, in the putative cancer-enhancing/immune-suppressive cluster, we observed an association between circulating M-MDSC and Tregs that might be explained by increase in Tregs induced by MDSC-derived immunosuppressive cytokines (16) as described previously in non-cancer settings (17, 18). NLR associated with M-MDSC and Tregs, which may reflect “emergency” myelopoiesis induced by tumor or by host-related conditions, that promotes production of not only classical myeloid cells such as neutrophils and monocytes but also myeloid-derived suppressor cells (19). In line with two inversely associated clusters of immune-based circulating biomarkers, we have previously shown a negative correlation between effector CD27− cytotoxic CD8+ T-cells and number of both CD33hi PMN-MDSCs and M-MDSC in pediatric cancer patients (19).

The current clinical trial was designed for patients with progressive, recurrent, or primarily metastatic high-risk tumors that are always heavily pretreated by prior multimodal anticancer therapy. Indeed, patients with measurable disease represented vast majority of cases enrolled to this clinical trial. Therefore, we may expect that patients evaluated in this clinical trial exhibit prior profound suppression of immune function. Indeed, the majority of sarcoma patients were lymphopenic. On peripheral blood immunograms, we showed distinct patterns of immune parameters such as prevailing CD8+ T-cell stimulation in patient KDO-0101 or marked immunosuppression in KDO-0114. However, observations from immunomonitoring and clinical course in the patient KDO-0101 are worth particular attention. In comparison to the rest of the study group, patient KDO-0101 exhibited a lymphocyte count within the reference range, a high proportion of effector T-cells, and low levels of all observed parameters associated with adverse disease outcome, namely, Treg count, M-MDSC count, and neutrophil-to-lymphocyte ratio. This DC-vaccinated patient experienced substantial regression of metastatic Ewing's sarcoma after the second relapse. In comparison to the initial DC vaccination, at DC rechallenge, a proportion of effector and activated DC increased, although ALC dropped. We also observed an increase in γδ T-cells, which may be attributable to therapy with zoledronic acid that was part of the third-line therapy prior to DC rechallenge. Zoledronic acid causes accumulation of isopentenyl pyrophosphates (IPP), leading to stimulation of γδ T-cells (20). γδ T-cells responding to zoledronic acid are Vγ9+Vδ2+ T-cells that sense IPP via Vδ2 TCR (20). Interestingly, however, in this patient, we observed an increase in number of Vγ9−Vδ2− T cells and depletion of Vγ9+Vδ2+ T-cells. It is of note that only in two out of nine pediatric sarcoma patients (KDO-0118 and KDO-0139), the Vγ9+Vδ2+ subset represented a majority of circulating γδ T-cells. This is an unexpected observation in the context of reported findings (21) and of our observations in adult carcinoma patients (7) and patients treated and evaluated in the DC clinical trial with non-sarcoma cancers (data not shown).

The second relapse in subject KDO-0101 occurred during maintenance therapy with DC ITx. The observed temporary regression of metastases of the Ewing's sarcoma after second relapse may have been related to the immune response induced by previous DC treatment. Despite stable disease on the third-line CTx topotecan/cyclophosphamide, the patient exhibited partial response after concomitant RTx and DC vaccination only. Performance status of the patient was good over a long period of time, namely, Karnofsky index over 80%, despite heavy metastatic involvement in skull, pelvic bones, spinal column, and liver. Performance status declined after 1 year of RTx, DCs ITx, and metronomic vinblastine and zoledronic acid. This unexpected observation suggests an opportunity to deliver such treatment to more patients. We observed substantial enhancement of T-cell reactivity toward DC-presented tumor antigens upon DC vaccination in patient KDO-0101 and to a lesser extent in four other sarcoma patients vaccinated with DCs and analyzed here. Thus, we confirmed that our anticancer DC-based vaccine stimulates a preexisting immune response against self-tumor antigens. Moreover, in the case of KDO-0101, functional *ex vivo* testing revealed that T-cell reactivity toward DC-presented self-tumor antigens persisted for a long period of time without DC treatment and was further boosted by DC rechallenge. In principle, the mechanism of action of anticancer DCs relies on stimulation of T-cell-mediated antitumor immune response targeting the presented cancer neoantigens. However, to date, the majority of patients treated with investigational DCs including the pediatric cancer patients in this clinical trial were end-stage or advanced cancer patients with extensive tumor mass and severely destroyed immune system. Limited clinical response achieved by DC-based ITx across numerous clinical trials can be attributed to both tumor-induced immunosuppression and, in heavily pretreated patients, also to anticancer therapy-induced immunosuppression. This is, nevertheless, supported by limited observational experience that enhancement of T-cell response to self-tumor antigens was related to the stage of the disease, that is, lower in cases with sarcomas in progression. It is thus crucial to overcome the immunosuppressive barrier to improve the efficacy of DC-based ITx as to have the antigen-presenting DC-based ITx combinable with cytokines, immune adjuvants, CTx, targeted therapy, and/or checkpoint inhibitors in order to boost T-cell effector functions and/or inhibit immune-suppressive pathways in the tumor mass (22). Ideally, selection of the right concomitant treatment to be combined with DC ITx shall be personalizable to target either particular immunosuppressive elements prevailing or particular immune effectors deficient in a particular patient, such as low-dose cyclophosphamide to deplete Tregs (23) or zoledronic acid to enhance γδ T-cells (24). In this context, immune-based biomarkers within the tumor microenvironment (if accessible) and/or systemic from peripheral blood could be exploited not only to provide an optimal ITx combination but also to select patients that would benefit from DC-based ITx. Regarding tumor-induced immunosuppression that is dependent on the tumor volume renders DC ITx less effective in patients with extensive tumor burden (25) and elicits higher tumor-specific immunologic response rates in the adjuvant compared to the metastatic setting (26). Thus, there is a rationale for the use of DC-based ITx earlier in the course of disease when tumor burden is still minimal; for example, in the adjuvant setting in patients at high risk of recurrence or in patients with minimal metastatic disease.

From our perspective beyond the study, anticancer DC vaccination could be more effective if appropriately personalized not only in terms of loading DC with self-tumor antigens but also in terms of (i) selection of the right patients that would benefit from ITx (such as patients with tumor with high mutational load), (ii) treatment at the right time when the disease and the level of immune suppression is minimal, and (iii) selection of right (possibly personalized) concomitant treatment that allows the optimal immunostimulation and anticancer activity of effector cells.

## Data Availability Statement

The datasets generated for this study are available on request to the corresponding author.

## Ethics Statement

The studies involving human participants were reviewed and approved by Ethics Committee, University Hospital Brno. Written informed consent to participate in this study was provided by the participants' legal guardian/next of kin or by the adult participants. Written informed consent was obtained from the minor(s)' legal guardian/next of kin for the publication of any potentially identifiable images or data included in this article.

## Author Contributions

LFe contributed to the study design, performed laboratory data acquisition and analysis, prepared figures, tables, supplementary material, contributed to data interpretation, and drafted the manuscript. PMu contributed to the trial design, performed patient enrollment and treatment, contributed to data interpretation, and drafted the manuscript. KP supervised IMP manufacturing, contributed to laboratory data acquisition and analyses, supplementary material preparation, and drafted the manuscript. IS performed statistical analysis, contributed to figure preparation, data interpretation, and drafted the manuscript. JM contributed to the trial design, participated in clinical and manufacturing data analysis, and drafted the manuscript. ZR performed PET/CT data acquisition, contributed to figure preparation, data interpretation, and drafted the manuscript. DV and EH contributed to the trial design, data interpretation, and revised the manuscript. DC participated in clinical data acquisition, contributed to supplementary material preparation, and revised the manuscript. LFa participated in clinical data acquisition and revised the manuscript. PMa and ZP contributed to the trial design, participated in patient treatment, and revised the manuscript. RD and JS contributed to the trial design, contributed to data interpretation, and revised the manuscript. LZ-D conceived the study design, designed and supervised laboratory data acquisition and analysis, contributed to data analysis and interpretation, and drafted and finalized the manuscript.

### Conflict of Interest

The authors declare that the research was conducted in the absence of any commercial or financial relationships that could be construed as a potential conflict of interest.
