# Supplementary material for: Assessment of Immune Response Following Dendritic Cell-Based Immunotherapy in Pediatric Patients With Relapsing Sarcoma
Source: Front Oncol. 2019 Nov 14;9:1169. doi: 10.3389/fonc.2019.01169 (PMC6868036; doi:10.3389/fonc.2019.01169)
Supplement: Supplementary file 1 [file Data_Sheet_1.PDF]

Supplemental material 1

Description of monitored circulating immune subsets

We performed immunomonitoring evaluating following circulating immune subsets: cytotoxic effector CD8+ T-cells (Eff CD8+), activated CD8+ T-cells (Act CD8+), natural killers (NK), NKT-like CD56+CD3+ T-cells (NKT-like), γδ T-cell (GD), regulatory T-cells (Tregs), monocytic myeloid-derived suppressor cells (M-MDSCs).

Cytotoxic CD8+ T cells play an important role in eradicating virus-infected cells and tumor cells. Naive CD8+ are activated by antigen-presenting DCs and by CD4+ T-cell co-stimulation, and then differentiate into antigen-specific effector cells with strong cytotoxic functions and the capacity to convert into memory CD8+ T-cells that persist for lengthy periods of time and but then rapidly proliferate and differentiate into secondary effector cells after recurrent antigen stimulation (Cui and Kaech, 2010).

NK cells are members of innate immunity that participate in early control against viral infection and in anti-tumor surveillance by their cytolytic functions and production of cytokines such as interferon-γ (IFN-γ) which boosts both adaptive and innate immune responses (Vivier et al., 2011). The production of IFN-γ helps to shape T-cell responses in lymph nodes, possibly by a direct interaction between naive T-cells and NK cells migrating to secondary lymphoid compartments (Martin-Fontecha et al., 2004), where the own presentation of antigen by dendritic cells (DC) to T-cells is reaching

NKT-like cells represent a minor lymphocyte population expressing determinants of both T-cells, such as TCR, and NK cells, such as inhibitory and activation receptors and some cytokine receptors that promote their effector function. NKT-like cells have been shown to mediate lysis of malignant cells and have been implicated in the control of tumor growth (Lu and Negrin, 1994;Baxevanis et al., 2003). The level of circulating NKT-like cells may relate to disease outcome in cancer patients (Zdrazilova-Dubska et al., 2012).

T-cell receptor (TCR) of mostly T-cells, such as helper T-cells and cytotoxic T-cells, is made up of 2 glycoprotein chains called alpha (α) and beta (β). Minor population of T-cells have on their surface distinct receptor formed by 2 chains called gamma (γ) and delta (δ), accordingly we call them γδ T-cells (or GD T-cells), and sense ligands that are fundamentally different from those recognized by classical αβ T-cells. γδ T-cells specifically recognize molecules over-expressed under stress conditions; Vδ2 T cells recognize small bacterial phosphoantigens, alkylamines and synthetic aminobisphosphonates, and Vδ1 T cells recognize stress-inducible MHC-related molecules MICA/B and several other ligands (Holtmeier and Kabelitz, 2005). γδ T-cells rapidly produce a variety of cytokines and exert potent cytotoxic activity against many types of tumor cells (Gober et al., 2003;Holtmeier and Kabelitz, 2005).

Regulatory T-cells engage in the maintenance of immunological self-tolerance by actively suppressing self-reactive lymphocytes (Hori et al., 2003). That leads to prevention of auto-immune diseases. It is obvious, that Tregs have immunosuppressive effect, which is related to down-regulation of anti-tumor defenses. A high absolute number of Tregs in cancer patients is related to poor prognosis (Wolf et al., 2003).

Myeloid-derived suppressor cells are considered to be strongly associated with tumor progression and contribute through immune and non-immune mechanisms to tumor promotion. MDSCs are a heterogeneous group of terminally undifferentiated myeloid cells of various origin (Talmadge and Gabrilovich, 2013;Pilatova et al., 2018) with monocytic MDSCs representing the majority of circulating MDSCs (Bronte et al., 2016;Pilatova et al., 2018). Their tumorigenic potential relies on the suppression of both innate and adaptive immune responses mediated by interference with trafficking of T-cells, induction of oxidative stress, release of immunosuppressive factors such as IL-10 and TGF-β, and promotion of immunosuppressive regulatory T-cells (Ostrand-Rosenberg and Sinha, 2009;Motallebnezhad et al., 2016).

Baxevanis, C.N., Gritzapis, A.D., and Papamichail, M. (2003). In vivo antitumor activity of NKT cells activated by the combination of IL-12 and IL-18. *J Immunol* 171**,** 2953-2959.

Bronte, V., Brandau, S., Chen, S.H., Colombo, M.P., Frey, A.B., Greten, T.F., Mandruzzato, S., Murray, P.J., Ochoa, A., Ostrand-Rosenberg, S., Rodriguez, P.C., Sica, A., Umansky, V., Vonderheide, R.H., and Gabrilovich, D.I. (2016). Recommendations for myeloid-derived suppressor cell nomenclature and characterization standards. *Nat Commun* 7**,** 12150.

Cui, W., and Kaech, S.M. (2010). Generation of effector CD8+ T cells and their conversion to memory T cells. *Immunological Reviews* 236**,** 151-166.

Gober, H.J., Kistowska, M., Angman, L., Jeno, P., Mori, L., and De Libero, G. (2003). Human T cell receptor gammadelta cells recognize endogenous mevalonate metabolites in tumor cells. *Journal of Experimental Medicine* 197**,** 163-168.

Holtmeier, W., and Kabelitz, D. (2005). gammadelta T cells link innate and adaptive immune responses. *Chem Immunol Allergy* 86**,** 151-183.

Hori, S., Nomura, T., and Sakaguchi, S. (2003). Control of regulatory T cell development by the transcription factor Foxp3. *Science* 299**,** 1057-1061.

Lu, P.H., and Negrin, R.S. (1994). A novel population of expanded human CD3+CD56+ cells derived from T cells with potent in vivo antitumor activity in mice with severe combined immunodeficiency. *J Immunol* 153**,** 1687-1696.

Martin-Fontecha, A., Thomsen, L.L., Brett, S., Gerard, C., Lipp, M., Lanzavecchia, A., and Sallusto, F. (2004). Induced recruitment of NK cells to lymph nodes provides IFN-gamma for T(H)1 priming. *Nat Immunol* 5**,** 1260-1265.

Motallebnezhad, M., Jadidi-Niaragh, F., Qamsari, E.S., Bagheri, S., Gharibi, T., and Yousefi, M. (2016). The immunobiology of myeloid-derived suppressor cells in cancer. *Tumour Biol* 37**,** 1387-1406.

Ostrand-Rosenberg, S., and Sinha, P. (2009). Myeloid-derived suppressor cells: linking inflammation and cancer. *J Immunol* 182**,** 4499-4506.

Pilatova, K., Bencsikova, B., Demlova, R., Valik, D., and Zdrazilova-Dubska, L. (2018). Myeloid-derived suppressor cells (MDSCs) in patients with solid tumors: considerations for granulocyte colony-stimulating factor treatment. *Cancer Immunol Immunother*.

Talmadge, J.E., and Gabrilovich, D.I. (2013). History of myeloid-derived suppressor cells. *Nat Rev Cancer* 13**,** 739-752.

Vivier, E., Raulet, D.H., Moretta, A., Caligiuri, M.A., Zitvogel, L., Lanier, L.L., Yokoyama, W.M., and Ugolini, S. (2011). Innate or adaptive immunity? The example of natural killer cells. *Science* 331**,** 44-49.

Wolf, A.M., Wolf, D., Steurer, M., Gastl, G., Gunsilius, E., and Grubeck-Loebenstein, B. (2003). Increase of regulatory T cells in the peripheral blood of cancer patients. *Clin Cancer Res* 9**,** 606-612.

Zdrazilova-Dubska, L., Valik, D., Budinska, E., Frgala, T., Bacikova, L., and Demlova, R. (2012). NKT-like cells are expanded in solid tumour patients. *Klin Onkol* 25 Suppl 2**,** 2S21-25.
