# Supplementary material for: Assessment of Immune Response Following Dendritic Cell-Based Immunotherapy in Pediatric Patients With Relapsing Sarcoma
Source: Front Oncol. 2019 Nov 14;9:1169. doi: 10.3389/fonc.2019.01169 (PMC6868036; doi:10.3389/fonc.2019.01169)
Supplement: Supplementary file 2 [file Data_Sheet_2.pdf]

Dendritic cell-based IMP manufacturing in Clean rooms

Dendritic cell vaccine manufacturing involved two phases – i) preparation of tumor lysate as a source of self-tumor antigens and ii) preparation of monocyte-derived DCs and their loading with tumor lysate.

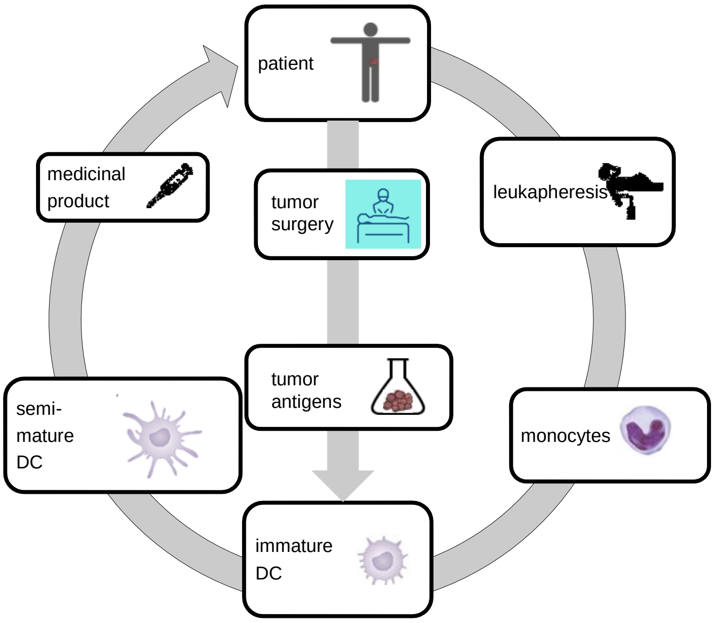


Tumor lysate was prepared as follows: a patient's tumor tissue of a volume at least 1 $cm^3$ was obtained during curative surgery or extended biopsy. In Clean rooms, necrotic areas and connective tissue were removed from the tumor tissue with a surgical scalpel keeping the specimen immersed in HBSS. The remaining tissue was minced into fragments of about 0.5 mm on each side by the use of a scalpel and forceps and crushed through the back of a syringe. Suspension of tumor fragments and cells in HBSS was lysed by repeated (5 times) freezing in liquid nitrogen and thawing at 37 °C. Obtained crude tumor lysate was centrifuged at 450 g /7 min /4°C to remove particulate components. The tumor lysate was released for DC manufacturing if the following criteria were met: i) the presence of viable tumor cells reported by a histopathologist, ii) protein concentration of 150 µg of total protein at minimum, iii) microbiologic sterility.

The leukapheretic product containing monocytes and lymphocytes was evalueated for the number of particular cell type. The numbers of leukocytes, B- and T-lymphocytes, monocytes and granulocytes in the leukapheretic product were evaluated hematology analyzer (XT-4000i, Sysmex) and flow cytometer (FC-500, Beckman Coulter) with staining for CD45, CD3, CD14 and CD19 (all antibodies from Beckman Coulter). Approximately $10^6$ cells were mixed with anti-CD45-PC7 (clone J33, 10 µl), anti-CD3-FITC (clone UCHT1, 10 µl), anti-CD14-PE (clone RMO52, 10 µl),

anti-CD19-PC5 (clone J3-119, 10 µl), incubated 20 min in dark, red blood cells were lyzed 15 min with 600 µl of VersaLyse (Beckman Coulter) and analyzed. Leukocytes were evaluated as CD45+, monocytes CD14+, T-lymphocytes CD3+, B-lymphocytes CD19+. Granulocytes were defined according to CD45 positivity and optical properties in side scattering. Monocytes for DC manufacturing were separated from leukapheretic product by elutriation or adherence to plastic surface. During elutriation (using Elutra cell separator, Gambro BCT), blood cells were separated on the basis of sedimentation velocity into six fractions and the last fraction rich for monocytes was used for DC manufacturing. Contaminating cells after elutriation were mainly granulocytes with similar sedimentation velocity to monocytes. Five hundred million monocytes of leukapheretic product adhered for 2 up to 4 hours in three 175 $cm^2$ tissue culture flasks with 35 mL of CellGenix® GMP DC Medium at 37 °C /5% $CO_2$, washed with HBSS and processed further.

Five hundred million monocytes seeded from elutriation product or attached by plastic adherence were cultivated in three 175 $cm^2$ tissue culture flasks with 70 mL of CellGenix® GMP DC medium supplemented with GM-CSF (1000 U/mL) and IL-4 (320 U/mL) at 37 °C / 5% $CO_2$ / 6 days. On day 3, fresh 70 mL of medium supplemented with the same concentration of GM-CSF and IL-4 was added to the culture. On day 6, immature DCs were exposed to autologous tumor lysate antigens (10 µg/mL) with keyhole limpet haemocyanin (KLH, 1 µg/mL), IL-4 (320 U/mL) and GM-CSF (1000 U/mL) at 37 °C/5% $CO_2$ for 1.5 to 2 hours. Maturation was induced by lipopolysaccharide (200 U/mL) and interferon-γ (50 ng/mL) an for additional 6 hours at 37 °C / 5% $CO_2$. Finally, semi-matured dendritic cells (smDCs) were collected using accutase (Accutase®, Corning), counted on Bürker cell chamber and frozen in aliquots of 2 x $10^6$ DCs in 100 µL of freezing medium CryoStor® CS2 or CS5 at -80 °C. All doses of DC-based investigational medical product were stored at -150 °C until administration to the patient.
