# Supplementary material for: Assessment of Immune Response Following Dendritic Cell-Based Immunotherapy in Pediatric Patients With Relapsing Sarcoma
Source: Front Oncol. 2019 Nov 14;9:1169. doi: 10.3389/fonc.2019.01169 (PMC6868036; doi:10.3389/fonc.2019.01169)
Supplement: Supplementary file 3 [file Data_Sheet_3.PDF]

**Subject No. / Primary diagnosis / Date and age at primary diagnosis**	**Disease course**	**Cancer therapy<br>(CTx. RTx. ITx.)**	**Stage of the disease and PS at DC ITx initiation / Date and age at the DC ITx initiation**	**Outcome<br>Stage of the disease in February 2019**
**KDO-0101**<br><br>Ewing sarcoma of the mandible<br>Mts parietal bone<br>EWS/FLI-1 pos<br><br>11/2011<br>10 yrs	1st relapse<br>Mts parietal bone<br>09/2015<br><br>2nd relapse 6/2017<br>Locoregional relapse with mts progression<br>Mts pelvis, spine, cranial bones, liver<br><br>3rd relapse<br>Metastatic progression<br>6/2018	1st line CTx: EuroEwing 08 12/2011 - 10/2012<br>Partial resection of the primary tumor mass in the mandible<br>1st PR 06/2012<br><br>HD CTx Treo/Mel + APBSCT 07/2012<br><br>RTx mandible and parietal bone 34 Gy + 45 Gy<br>09/2012 - 11/2012<br><br>Euro Ewing maintenance CTx 7 x VAC 10/2012-5/2013<br>1st CR<br><br>Mts resection parietal bone 09/2015<br>2nd CR<br><br>2nd line CTx: VCR/Irino + Pazopanib 09/2015 – 04/2016<br><br>RTx cranial bones, total dose 41 Gy 1/2016- 2/2016<br><br>HD CTx Treo/Mel + APBSCT 04/2016<br><br>CYC 9/2016 – 06/2017<br><br>Experimental DC immunotherapy 1st course 20 doses<br>08/2016 – 06/2017<br><br>Resection of the mandible 06/2017<br><br>3rd line CTx: Topo/CYC + Zoledronic acid 07/2017 - 09/2017<br>SD	2nd CR<br>Karnofsky 100<br><br>08/2016<br>15 yrs	Exitus letalis 11/2018<br>17 yrs<br><br>Metastatic progression of Ewing sarcoma

		RTx mandible cranial bones pelvis, total dose 21Gy<br>08/2017 - 09/2017<br><br>4th line CTx: VBL weekly + Metformin 01/2018 – 06/2018<br>SD<br><br>Nivolumab every 2 weeks 02/2018- 06/2018<br><br>Experimental DC immunotherapy 2nd course 03/2018 – 11/2018, PD 06/2018		
**KDO-0102**<br><br>Localized high-grade osteosarcoma of the right distal femur<br><br>05/2012<br>6 yrs	1st relapse<br>Mts lungs and left tibia<br>4/2014<br><br>2nd mts progression<br>Mts lungs bilat.<br>11/2015<br><br>3rd mts progression<br>Lungs bilat, thorax, abdomen<br>6/2016	1st line CTx: AOST 0331 05/2012 - 02/2013<br><br>Resection - primary tumor 08/2012, 1st CR<br><br>Mifamurtid 10/2012 - 04/2013<br><br>2nd line CTx: Ifo/Eto +Zoledronic acid 06/2014 - 01/2015<br>PR<br><br>3rd line CTx: COMBAT III + Pazopanib 01/2015 - 09/2015<br>PD<br><br>4th line CTx: re-challenge therapy 2 blocks HD MTX (12 g/m^2)<br>10/2015-11/2015<br>PD<br><br>5th line CTx VBL /CYC + Sunitinib 12/2015 - 02/2016<br><br>RTx lungs and left calf total dose<br>15Gy + 36Gy 01/2016 -02/2016<br><br>Nivolumab 4 doses 03/2016-04/2016 PD<br><br>RTx lungs total dose:17.5Gy 06/2016<br><br>Experimental DC immunotherapy 17 doses<br>01/2016-09/2016	PD<br>Lansky 80<br><br>01/2016<br>10 yrs	Exitus letalis 10/2016<br>11 yrs<br><br>Metastatic progression of osteosarcoma

**KDO-0114**<br><br>Synovial sarcoma of the left thigh, Mts lungs, retroperitoneum<br><br>12/2014<br>13 yrs	1st metastatic progression left hemithorax 10/2016	1st line CTx<br>ARST 1321 + Metformin + Pazopanib<br>12/2014 - 05/2015<br><br>RTx primary tumor 45Gy 03/2015<br><br>Resection of the primary tumor 05/2015<br><br>COMBAT IIII 05/2015 -5/2016<br><br>Thoracoscopic extirpation of left hemithorax Mts 10/2016<br><br>2nd line CTx ARST08P1, 1 block IE 12/2016<br><br>Palliative RTx left hemithorax 15Gy 01/2017<br><br>Paliative experimental Tx<br>Valproate/Metformin/Lipanthyl/Sunitinib 12/2016 - 06/2017<br><br>Experimental DC immunotherapy 11 doses 01 - 06/2017	PD<br>Karnofsky 80<br><br>01/2017<br>15 yrs	Exitus letalis 07/2017<br>16 yrs<br><br>Metastatic progression of synovialo-sarcoma
**KDO-0118**<br><br>Localized Ewing sarcoma of the spine C5-Th2<br>Extradural and intraspinal involvement<br>EWS/FLI-1 neg<br><br>06/2005<br>13 yrs	1st locoregional relapse paraspinal right C6-Th2 08/2016<br>EWSR1 gene disruption confirmed<br><br>2nd locoregional progression 04/2017	1st line CTx Euro Ewing 99, 07/2005 – 12/2005<br><br>1st CR<br>HD CTx + APBSC 01/2006<br>RTx - primary tumor 49.2Gy 03/2006 - 04/2006<br><br>2nd line CTx AEWS1031, 08/2016 - 02/2017, 2 blocks VTC – SD, 2 blocks of VCR/IRINO<br>PR	PR<br>Karnofsky 100<br><br>02/2017<br>24 yrs	Exitus letalis 08/2017<br>25 yrs<br><br>Locoregional progression of Ewing sarcoma
**KDO-0119**<br><br>Alveolar rhabdomyosarcoma		1st line CTx ARST 08P1 + TEM 10/2016 -05/2018<br><br>Maintenance CTx VNR+ CYC (EpSSG RMS 2005)<br>05/2018 – ongoing	PR<br>Karnofsky 80<br><br>04/2018	Alive<br><br>1st CR

Primum ignotum<br>Mts bones and bone marrow<br><br><br>10/2016<br>12 yrs		Experimental DC immunotherapy 22 doses 4/2018 – ongoing	13 yrs	
**KDO-0124**<br><br>Localized osteoblastic osteosarcoma of the right proximal tibia<br><br>12/2012<br>14 yrs	1st relapse<br>Mts lungs bilat.<br>09/2014<br><br>2nd relapse<br>Mts lungs bilat.<br>01/2017	1st line CTx AOST 0331<br>12/2012 - 11/2013<br><br>Resection primary tumor 03/2013<br>1st CR<br><br>Resection – lung Mts 11/2014<br>2nd CR<br><br>2nd line CTx 4 x Ifo/Eto 02/2015 - 04/2015<br>COMBAT III 05/2015 - 01/2017<br><br>Resection of the lung Mts 02/2017<br><br>3rd line CTx AOST 1321 + VBL+ CYC 02/2017 - 10/2017<br><br>Experimental DC immunotherapy 16 doses 05/2017-12/2017	2nd mts relaps<br>PR after resection<br>Karnofsky 100<br><br>05/2017<br>19 yrs	Exitus letalis 1/2018<br>19 yrs<br><br>Progression of osteosarcoma
**KDO-0131**<br><br>Embryonal rhabdomyoma sarcoma of the pelvis<br><br>Infiltration of the prostate, seminal tubes, rectum, ext. and int. obturator		1st line CTx EpSSG 2005 09/2017 – 06/2018<br><br>Experimental DC immunotherapy 18 doses<br>06/2018 – ongoing	PR<br>Karnofsky 70<br><br>06/2018<br>19 yrs	Alive<br><br>PR

muscle, penis, skeleton<br><br>08/2017<br>18 yrs				
**KDO-0133**<br><br>Localized osteosarcoma of the right proximal femur<br><br>10/2011<br>17 yrs	1st relapse 06/2015<br>Lung Mts<br><br><br>2nd relapse<br>Lung Mts<br>06/2016<br><br>3rd relapse<br>Lung Mts<br>01/2018<br><br>4th relapse<br>Lung Mts<br>12/2018	1st line CTx AOST 0331<br>10/2011 – 07/2012<br><br>Resection of th primary tumor 01/2012<br>1st CR<br><br>RTx primary tumor 54Gy 02/2012<br><br>Resection of lung Mts 06/2015<br>2nd CR<br><br>Resection of lung Mts 06/2016<br>3rd CR<br><br>2nd line CTx AOST 1321 08/2016 – 12/2017 + Sunitinib from 11/2016 + Metformin from 10/2017<br><br>RTx lungs 15Gy 02/2017-03/2017<br><br>Resection lung Mts 02/2018<br>4th CR<br><br>3rd line CTx COMBAT III 04/2018 – 12/2018<br><br>Experimental DC immunotherapy 1st course 6 doses 06/2018 - 09/2018, 2nd course 6 doses 12/2018- 2/2019<br><br>Resection lung Mts 12/2018<br>5th CR	PD<br>Karnofsky 100<br><br>06/2018<br>24 yrs	Alive<br><br><br>PD<br>Karnofsky 90
**KDO-0139**	1st relapse<br>Mts lungs bilat.	1st line CTx AOST0331 09/2016 – 05/2017	SD<br>Karnofsky 90	Alive

High-grade osteoblastic osteosarcoma of the left distal femur<br>Solitary lung Mts at diagnosis<br><br>09/2016<br>20 yrs	05/2018	Resection of the primary tumor 12/2016<br>1st CR<br><br>Resection lung mts 07/2018<br><br>2nd line CTx AOST0331, 07/2018<br>PR after 2 blocks<br>SD<br><br>RTx lung Mts 15Gy 08/2018<br><br>3rd line CTx COMBAT III modif 10/2018- 02/2019 + Olaparib 01/2019 - 02/2019<br><br>Experimental DC immunotherapy 6 doses 11/2018 – 02/2019	11/2018<br>22 yrs	SD<br>Karnofsky 90

Table 1. The diagnosis, clinical course, cancer therapy and outcome of 9 patients with sarcoma treated by dendritic cell-based immunotherapy.

In the columns (from left to right) are described 1/ The number (No.) of subject (patient), the primary diagnosis, date and age of primary diagnosis. 2/ The clinical course of the disease. 3/ Cancer therapy, CTx – chemotherapy, RTx – radiotherapy, ITx – immunotherapy. 4/ The stage of the disease and performance status (PS) at dendritic cell (DC) ITx initiation, the date and age at DC ITx inititation. 5/ Outcome and stage of the disease in February 2019. APBSC – Autologous Peripheral Blood Stem Cell transplantation, Bilat – bilateral, CR – complete response[1)], CYC – cyclophosphamide, Doxo - doxorubicin, Eto – Etoposide, EWS/FLI-1 - a tumor-specific 11;22 translocation (1), Ext – external, HD – high dose, Ifo/Eto – chemotherapy block – ifosfamide, etoposide, Int – internal, Irino – irinotecan, IRS – intergroup rhabdomyosarcoma study, M – month, Mts – metastasis or metastases, mts – metastatic, MTX – methotrexate, neg – negative, No. – number, PD – progressive disease[2)] pos – positive, PR – partial remission[3)], PS – performance status (Lansky/Karnofsky), SD – stable disease[4)], TEM – temsirolimus, Temo – temozolomide, Tx – therapy, Topo/CYC - chemotherapy block – topotecan, cyclofosfamide, Treo/Mel – high-dose chemotherapy regimen – treosulfan and melphalan, VAC – chemotherapy block - Vincristine, Actinomycin, Cyclophosphamide, VBL – vinblastin, VCR – vincristine, VCR/Irino – chemotherapy block – vincristine, irinotecan, VTC – Chemotherapy block – Vincristine, Topotecan, Cyclofosfamide, yrs – years, **Chemotherapy – protocols:** AEWS1031 protocol (Ewing sarcoma) – combination of vincristine, doxorubicin, cyclophosphamide, ifosfamide, etoposide, AOST 0331 protocol (osteosarcoma) – combination of cisplatin, doxorubicin, methotrexate, AOST 1321 protocol (osteosarcoma) – a repeated dose of denosumab = RANKL inhibitor, ARST 08P1 protocol (rhabdomyosarcoma) – combination of vincristine, irinotecan, ifosfamide, etoposide, doxorubicin, cyclophosphamide, actinomycin, ARST 1321 protocol (non-rhabdomyosarcoma soft tissue sarcomas) - combination of ifosfamide, doxorubicin and pazopanib, COMBAT III - long term metronomic treatment based on a combination of celecoxib, etoposide, temozolomide, fenofibrate, ergocalciferol,

bevacizumab, vinorelbine, cis-retinoic acid, EpSSG 2005 protocol (rhabdomyosarcoma) – combination of ifosfamide, vincristine, actinomycin, doxorubicin, EuroEwing2008 protocol (Ewing sarcoma) – combination of vincristine, ifosfamide, doxorubicin, etoposide, actinomycin, cyclophosphamide, RECIST 1.1 The Response Evaluation Criteria in Solid Tumors, revision 2009 (2): *Complete Response* (CR): Disappearance of all target lesions. Any pathological lymph nodes (whether target or non-target) must have reduction in short axis to < 10 mm. *Progressive Disease* (PD): At least a 20% increase in the sum of the diameters of target lesions, taking as reference the smallest sum on study (this includes the baseline sum if that is the smallest on study). In addition to the relative increase of 20%, the sum must also demonstrate an absolute increase of at least 5 mm. (Note: the appearance of one or more new lesions is also considered progressions). *Partial Response* (PR): At least a 30% decrease in the sum of the diameters of target lesions, taking as reference the baseline sum diameters. *Stable Disease* (SD): Neither sufficient shrinkage to qualify for PR nor sufficient increase to qualify for PD, taking as reference the smallest sum diameters while on study.

Reference:

1. WA May; SL Lessnick; BS Braun; M Klemsz; BC Lewis; LB Lunsford; R Hromas; CT Denny. The Ewing's sarcoma EWS/FLI-1 fusion gene encodes a more potent transcriptional activator and is a more powerful transforming gene than FLI-1. *Mol Cell Biol* 13, 7393–8 (1993)
2. LH Schwartz; S Litière; E de Vries; R Ford; S Gwyther; S Mandrekar; L Shankar; J Bogaerts; A Chen; J Dancey; W Hayes; FS Hodi; OS Hoekstra; EP Huang; N Lin; Y Liu; P Therasse; JD Wolchok; L Seymour. RECIST 1.1—Update and clarification: From the RECIST committee. *Eur J Cancer* 62, 132–137 (2016)
