# Supplementary material for: Assessment of Immune Response Following Dendritic Cell-Based Immunotherapy in Pediatric Patients With Relapsing Sarcoma
Source: Front Oncol. 2019 Nov 14;9:1169. doi: 10.3389/fonc.2019.01169 (PMC6868036; doi:10.3389/fonc.2019.01169)
Supplement: Supplementary file 4 [file Data_Sheet_4.PDF]

Date	Dose No.	ALC	NLR	M-MDSC	Ef CD8+	Act CD8+	NK	NKT-like	Treg	GD	TcRGD γ9-δ2+	TcRGD γ9+δ2+	TcRGD γ9+δ2-	TcRGD γ9-δ2-	γ9-δ2+ CD314		γ9+δ2+ CD314		γ9+δ2- CD314		γ9-δ2- CD314	
															+	-	+	-	+	-	+	-
		10^6/ml	ratio	10^6/ml	%	%	%	%	%	%	%	%	%	%	%	%	%	%	%	%	%	%
29.8.2016	bas	1.9	0.8	0.07	73.8	34.8	1.6	4.9	5.9	2.9	9	33	20	39	72	28	7	93	6	94	79	21
12.9.2016	d 1	1.6	1.7	0.06	74.8	38.5	1.5	4.8	5.3	3.2	6	33	16	44	57	43	11	89	6	95	71	29
3.10.2016	d 2	1.4	1.2	0.06	69.9	32.3	0.9	5.3	5.5	3.5	6	31	19	44	79	21	42	59	19	81	81	19
17.10.2016	d 3	1.6	1.8	0.09	73.3	23.2	1.0	5.3	7.2	3.6	11	26	20	43	80	20	44	57	26	74	76	24
31.10.2016	d 4	1.5	1.4	0.12	73.4	28.4	1.0	6.4	4.3	4.2	10	25	21	44	88	12	39	61	41	59	85	15
14.11.2016	d 5	1.3	3.6	0.29	66.7	23.2	0.9	6.2	5.3	2.8	11	16	11	62	67	33	28	72	25	75	80	20
28.11.2016	d 6	1.4	1.2	0.07	58.3	21.7	0.5	5.1	4.5	3.2	8	25	22	46	83	17	41	60	34	66	76	24
12.12.2016	d 7	1.3	1.8	0.07	73.7	22.1	0.8	5.7	5.8	3.5	8	19	21	52	86	14	23	77	13	88	82	18
29.12.2016	d 8	1.0	2.8	0.16	76.3	22.5	0.8	5.2	4.6	3.8	8	19	27	46	91	9	52	48	36	64	81	19
16.1.2017	d 9	1.2	2.7	0.06	64.8	19.5	0.8	5.8	5.7	3.9	7	16	25	53	73	27	16	84	8	92	77	23
30.1.2017	d 10	1.2	2.0	0.05	68.2	17.3	0.8	5.3	4.3	4.0	11	16	21	52	70	30	37	63	14	87	67	33
13.2.2017	d 11	1.4	1.7	0.04	62.7	78.6	0.7	4.6	6.1	3.7	9	12	22	57	75	25	10	90	11	89	81	19
27.2.2017	d 12	1.4	2.2	0.12	68.1	26.2	1.3	5.6	8.0	4.1	9	11	24	56	83	17	17	83	6	94	80	20
13.3.2017	d 13	1.3	1.6	0.12	68.1	11.9	1.0	5.1	4.6	4.1	9	13	21	56	70	30	26	74	7	93	75	26
27.3.2017	d 14	1.6	2.2	0.16	75.1	21.6	1.4	6.4	5.5	3.4	10	17	29	44	86	14	21	80	4	96	84	16
10.4.2017	d 15	1.2	2.5	0.11	78.3	25.4	1.5	6.5	4.5	4.0	11	18	22	50	68	32	17	83	5	96	69	31
24.4.2017	d 16	1.4	1.8	0.09	70.0	28.0	1.3	5.9	4.7	4.0	11	16	25	49	73	27	17	83	8	92	75	25
15.5.2017	d 17	1.2	2.5	0.14	69.4	19.0	0.9	6.3	5.0	4.2	8	16	23	53	76	24	78	22	65	35	81	19
29.5.2017	d 18	1.4	1.8	0.13	77.1	31.6	1.1	5.3	3.9	3.8	12	8	28	52	50	50	52	48	37	64	56	44
12.6.2017	d 19	1.3	2.5	0.12	71.2	39.4	1.2	5.9	4.7	5.1	11	4	27	58	73	27	77	23	47	53	74	26
14.3.2018	bas re	1.0	1.6	0.08	1.6	48.9	3.3	3.1	6.7	6.4	7	0	19	74	80	20	ND	ND	81	19	79	21
28.3.2018	re 1	0.8	2.4	0.07	2.5	32.1	4.1	3.9	5.5	6.2	6	1	18	75	67	33	ND	ND	66	34	89	11
11.4.2018	re 2	1.2	1.4	0.20	1.6	38.0	3.5	3.9	5.5	5.6	4	0	23	73	92	8	ND	ND	74	26	78	22
25.4.2018	re 3	1.2	1.8	0.23	2.0	57.3	3.7	3.6	7.3	6.2	3	0	21	76	71	29	ND	ND	66	34	75	25
9.5.2018	re 4	1.2	2.0	0.20	2.2	28.7	3.7	3.5	6.1	5.6	7	0	19	74	87	13	ND	ND	49	51	88	12
23.5.2018	re 5	0.7	4.6	0.37	5.0	32.4	3.9	4.5	5.5	5.8	5	0	28	68	77	23	ND	ND	65	35	86	14
6.6.2018	re 6	0.6	3.9	0.21	4.1	29.9	3.4	4.7	5.2	5.8	5	0	22	73	73	27	ND	ND	37	63	77	23
20.6.2018	re 7	1.9	4.3	0.83	5.1	20.2	1.3	1.6	5.8	1.6	6	0	18	76	100	0	ND	ND	28	72	74	26
4.7.2018	re 8	0.9	3.1	0.48	3.6	13.1	0.4	2.5	6.9	1.4	12	0	21	67	100	0	ND	ND	8	92	90	10
18.7.2018	re 9	1.4	3.3	0.39	3.7	24.1	1.1	3.0	4.8	4.5	5	1	23	71	67	33	ND	ND	0	100	84	16
15.8.2018	re 10	1.5	1.0	0.13	1.1	72.8	3.1	6.8	6.0	8.2	5	0	30	65	89	11	ND	ND	13	87	82	18

Levels of circulating immune markers at each dose of both lines of DC-based therapy in subject KDO-0101: Date – date of the peripheral blood collection; Dose No. – the order of DC-based immunotherapy (ITx) dose after which the sample of peripheral blood was collected, bas – baseline; d 1-19 – dose 1-19. Values at DC rechallenge are shown in yellow: re – rechallenge dose 1-10; bas re – baseline before restart of DC-treatment = rechallenge ALC – absolute lymphocyte count; NLR – neutrophil-to-lymphocyte ratio; eff CD8+ - circulating effector cytotoxic T-cells (% of T-cells); act CD8+ - activated cytotoxic T-cells (% of HLA-DR+ of CD8+ T-cells); NK – circulating natural killers (% of lymphocytes); NKT-like – circulating NKT-like cells (% of lymphocytes); GD-T – gamma-delta T-cells (% of lymphocytes); Treg – regulatory T-cells (% of CD4+ T-cells); M-MDSC – monocytic myeloid-derived suppressor cells. TcRGD – gamma-delta receptor of T-cells. 4 variants of TcRGD (γ9-δ2+, γ9+δ2+, γ9+δ2-, γ9-δ2-) are shown in color (in the middle), in each variatn is shown the proportion (%) of CD314 positivity (+) and negativity (-) (in the right).
